# Supplementary material for: Virtual Screening Technology for Two Novel Peptides in Soybean as Inhibitors of α-Amylase and α-Glucosidase
Source: Foods. 2023 Dec 6;12(24):4387. doi: 10.3390/foods12244387 (PMC10743026; doi:10.3390/foods12244387)
Supplement: Supplementary file 1 [file foods-12-04387-s001.zip › foods-2731501-supplementary.pdf]

Supplementary data

Virtual screening technology for two novel peptides in soybean as inhibitors of α-amylase and α-glucosidase

Calculate the binding energy through the following function:

$$\Delta G_{Bind}=C_{lipo\text{-}lipo}\sum f(r_{lr})+C_{hbond\text{-}neut\text{-}neut}\sum g(\Delta r)h(\Delta a)+C_{hbond\text{-}neut\text{-}chargedt}\sum g(\Delta r)h(\Delta a)$$

$$+C_{hbond\text{-}charged\text{-}charged}\sum g(\Delta r)h(\Delta a)+C_{max\text{-}metal\text{-}ion}\sum f(r_{lm})$$

$$+C_{rotb}H_{rotb}+C_{poral\text{-}phob}V_{poral\text{-}phob}+C_{coul}E_{coul}+C_{vdW}E_{vdW}+\text{solvation terms}$$

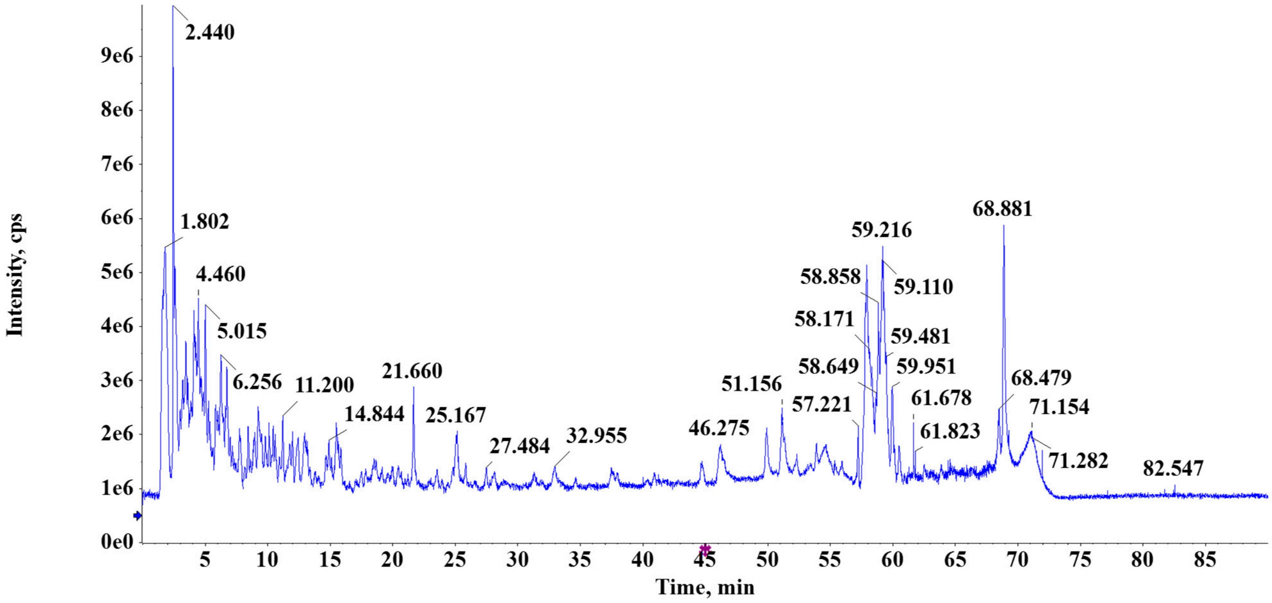

Figure S1. Total ion chromatogram of soybean peptides

Table S1. Detailed information of 202 peptides used for virtual screening

NO.	Sequence	PSMs	IonScore	M+H [Da]	ΔM [ppm]	RT [min]	Binding Energy [kcal/mol]	
							α-Amylase	α-Glucosidase
1	AIVII	60	43.33	528.376	1.706	31.367	-7.1	-6
2	VSILDTN	24	42.76	761.404	0.619	15.483	-6.9	-6.1
3	VPLPAG	16	39.52	553.335	1.238	10.933	-8.5	-7.2
4	KSNILE	16	41.61	703.398	-1.244	7.883	-7.6	-6.6
5	VPLPQ	16	41.77	553.335	1.256	10.933	-8.1	-7.5
6	GNQEQE	14	40.73	704.285	0.192	2.433	-7.2	-7.3
7	SAEFGSL	14	40.84	710.333	-2.958	21.933	-7.9	-7.1
8	LDQNPR	14	41.5	742.384	-0.367	3.283	-8.2	-6.2
9	VFVIPA	14	44.3	645.398	2.080	29.450	-8.6	-7.1
10	GNQEGAE	13	40.73	704.285	0.178	2.433	-6.8	-6.7
11	NEGDVL	13	42.05	646.305	1.806	11.850	-7	-7.1
12	PVDTVVA	12	40.83	700.390	2.683	18.350	-7.9	-6.8
13	mTGGIA	12	47.48	565.274	15.378	5.650	-7.3	-5.3
14	DFVLDNE	12	48.48	851.377	-1.192	22.150	-7.8	-7.2
15	mDNLQSK	11	43.79	851.377	-18.176	22.217	-7.6	-6.8
16	RLNLLTL	10	39.95	842.546	-0.399	31.567	-7.9	-6.7
17	mDNIITE	10	40.05	851.377	-5.496	22.133	-7.6	-6
18	SSPDIYNPQ	10	40.55	1020.464	0.738	12.817	-8.2	-6.6
19	LDQTPRVF	10	42.26	975.525	-1.379	18.333	-9.1	-7.6
20	GHAPISLPNQL	10	45.19	1146.625	-1.366	26.017	-8.2	-6.6
21	ISPLPVLKE	10	45.27	995.614	0.438	25.383	-8.4	-5.7
22	AENNQRNF	10	47.41	992.455	0.365	4.633	-7.6	-7.1
23	ADDVL	10	47.52	532.261	-1.323	12.200	-7.8	-7.1
24	DTSNFNNQLDQ NPRVF	9	40.09	1908.883	-0.398	26.233	-7.5	-5.5
25	mNDLLTE	9	42.11	851.377	-5.156	22.150	-7	-6.4
26	GDVVEYNPQ	9	43.96	1020.462	-1.519	12.700	-8.2	-6.9
27	ADVVSI	9	45.19	603.336	2.695	24.967	-7.5	-6.4
28	LAGNPDIEYPET m	9	46.32	1465.653	0.804	20.300	-8.4	-5.8
29	LAGNPDIEHPE	9	57.33	1191.564	-0.490	11.100	-8.2	-6.9
30	DTSNFNNQLDQ TPRVF	9	63.06	1895.887	-0.681	29.250	-7.7	-6.6
31	VPIPGA	8	39.52	553.335	1.238	10.933	-8.5	-6.6
32	VAGAL	8	39.81	430.266	-0.709	13.683	-7.7	-5.3
33	SLDFPAL	8	40.54	762.403	-0.166	37.400	-9	-7.2
34	NIVSISKP	8	42.8	857.509	0.136	14.833	-7.6	-6.1
35	NISVLNVV	8	43.09	857.509	0.136	14.833	-8.2	-6.4

36	EAVmIAL	8	43.78	762.405	-2.198	37.500	-7.4	-6.2
37	GNPDIEHPE	8	44.46	1007.442	-0.451	7.250	-8.3	-5.9
38	NNQLDQNPRVF	8	44.66	1344.666	0.524	18.300	-8.1	-7.1
39	RKQEEDEDEEQ QRE	8	45.1	1847.796	-2.196	2.767	-6.7	-5.7
40	IAQAQEI	8	45.83	772.431	14.282	6.650	-8.1	-6.4
41	GDLIAVPT	8	47.1	785.440	-0.952	25.733	-8.3	-6.6
42	AEFGSL	8	47.41	623.304	1.072	21.550	-7.7	-6.4
43	VVTLDTN	8	49.58	761.403	-1.288	15.517	-6.9	-6.2
44	GGLSVISPK	8	58.59	857.511	1.658	14.567	-7.7	-6.4
45	AESFGSI	7	39.75	710.333	-2.958	21.933	-7.6	-7.1
46	TSLDFPAL	7	39.88	863.451	-0.052	37.483	-9.3	-6.4
47	ASEGFSI	7	40.84	710.333	-2.958	21.933	-8	-6.4
48	LAGNPDIEHPET m	7	42.96	1439.646	-0.709	11.383	-7.5	-7.1
49	DLQDILK	7	44.45	844.477	-0.976	22.317	-7.5	-6.3
50	LAPIDTKPQ	7	44.6	982.556	-0.335	9.700	-7.8	-6.9
51	NTGDEPVVAI	7	50.35	1014.510	-0.098	23.583	-7.6	-6.6
52	GNPDIEHPETm	7	51.75	1255.525	-0.586	7.683	-7.4	-6
53	LDQNPRVF	7	51.85	988.519	-1.870	15.400	-8.5	-8.1
54	LDKGIGTII	7	52.45	929.567	0.021	24.717	-7.8	-5.4
55	LGGAIPEE	6	39.68	785.404	-0.023	15.633	-7.5	-6.4
56	IRLTL	6	39.87	615.418	-1.973	20.233	-8.1	-6.7
57	TNPLTQ	6	39.92	673.353	1.454	6.400	-7.6	-6.6
58	SKNILE	6	39.94	703.399	0.289	7.833	-7.1	-6.7
59	NFNNQLDQNPR VF	6	41.64	1605.775	-1.485	24.800	-7.4	-6.1
60	LNALPEE	6	42.33	785.404	-0.011	15.633	-8.6	-7.3
61	GADLLVE	6	42.46	716.383	0.864	17.733	-8.1	-6.6
62	SEDKPFN	6	43.03	836.378	-0.974	5.833	-8.2	-7.4
63	GENALPR	6	43.73	756.398	-2.547	5.583	-8.1	-6.9
64	FVVLPA	6	44.22	645.398	1.113	29.417	-8.1	-7.2
65	KEQQQEQQQEE QPLEVR	6	45	2154.042	-0.035	9.250	-6.7	-5.2
66	AIPVNKPGRF	6	45.52	1098.641	-0.869	11.483	-9.4	-6.5
67	SYDTKFEE	6	46.1	1018.437	0.141	10.633	-8.2	-7.4
68	LVLAGH	6	47	609.370	-3.151	6.717	-7.7	-7
69	VVLPVQNDGST GL	6	48.62	1298.696	0.514	27.083	-8.5	-5.8
70	PPNPHIGIN	6	48.86	958.511	0.041	12.833	-8.9	-7
71	NNQLDQTPRVF	6	50.35	1331.670	0.120	21.383	-7.9	-6.9
72	VGQDIQSK	6	50.58	874.463	0.130	3.933	-7.7	-6.5
73	ISSEDKPFN	6	53.49	1036.495	0.022	8.867	-7.9	-5.8

74	DFVLDNEGNPLE	6	54.52	1361.621	-0.932	32.883	-7.6	-6.3
75	NLRLITL	5	39.95	842.546	-0.399	31.567	-7.9	-6.7
76	EVEGPL	5	40.85	643.322	-12.256	27.250	-7.7	-6.7
77	EVGEPL	5	40.85	643.322	-12.256	27.250	-7.7	-6.6
78	TTDETLAL	5	41.33	863.449	15.927	37.333	-8	-6.7
79	GLDLVGS	5	42.2	660.356	0.055	20.517	-7.7	-6.6
80	QDLLVE	5	42.46	716.383	0.878	17.733	-7.2	-6.3
81	KGGLVL	5	42.61	586.393	0.767	8.467	-7	-5.7
82	FPADGT	5	43.16	607.272	0.199	10.150	-8.5	-8
83	GDAPNNmRQF	5	46.54	1165.505	-0.771	6.450	-9.2	-6.5
84	ADGNYFVPK	5	47.14	1010.504	9.394	7.000	-9.6	-6.3
85	SVLYVSLG	5	47.44	837.483	13.422	11.450	-8.8	-7.1
86	VDVASI	5	48.03	603.336	2.446	25.050	-7.6	-5.7
87	VDSVDI	5	48.29	647.326	2.156	19.700	-7.7	-7.1
88	NFNNQLDQTPRVF	5	48.49	1592.778	-2.136	27.533	-8.3	-7.9
89	YVVNPDNNE	5	49.9	1063.469	0.032	10.017	-8.6	-7.2
90	VLPVQNDASTGL	5	50.21	1213.642	-0.252	22.867	-7.7	-7
91	AGDGDVL	5	55.87	646.305	1.066	11.883	-7.2	-6.8
92	ADGGDVI	5	55.87	646.305	1.066	11.883	-6.8	-6.4
93	WNILGK	4	39.79	730.412	-16.769	15.967	-8.5	-7.2
94	QGALPmGF	4	39.86	836.396	-0.837	19.467	-8.7	-7.6
95	PESIAVGL	4	39.86	785.441	1.287	25.800	-7.9	-6.8
96	RVAGIITL	4	39.95	842.546	-0.411	31.567	-8.2	-4.9
97	RGGILLTL	4	39.95	842.546	-0.411	31.567	-8.6	-6.4
98	GNVIIITL	4	39.95	842.546	12.929	31.567	-8	-6.1
99	RNILLTL	4	39.95	842.546	-0.399	31.567	-7.8	-6.5
100	SRNLIE	4	40	731.405	-0.216	8.533	-8	-7.3
101	HADADYL	4	40.19	804.352	-0.012	11.150	-8.5	-7.7
102	ARARPS	4	40.26	657.368	-16.789	4.083	-8.2	-7.4
103	PSKAQEL	4	40.34	772.431	13.667	6.633	-7.9	-6.9
104	PSQAKEL	4	40.34	772.431	13.667	6.633	-8.5	-7
105	SPPLTPSSIH	4	40.5	1035.547	0.266	16.100	-8.9	-7.3
106	ESVFPAL	4	40.5	762.403	-0.166	37.400	-8.4	-6.8
107	ISKEQIRAL	4	40.68	1057.635	-1.030	11.783	-7.3	-6.4
108	DVSVDI	4	40.94	647.326	2.156	19.700	-8.4	-7
109	SRNPIYSN	4	41.04	950.468	-0.808	6.917	-8.7	-7.5
110	FREGDLIAVPT	4	41.12	1217.652	-0.533	27.550	-8.1	-6.1
111	ALIVPS	4	41.19	599.377	1.752	18.283	-8.5	-6.3
112	ENGVLGA	4	41.58	659.338	2.884	5.083	-7.5	-7.8
113	NELDKGIGTII	4	41.72	1172.653	0.766	29.567	-7.8	-6

114	FEmARANEE	4	42	1112.457	-9.598	14.500	-8.6	-7.3
115	LDISES	4	42.57	663.323	5.202	11.683	-7.4	-6.4
116	KNLVI	4	42.61	586.393	0.784	8.467	-7.7	-6.5
117	VEGGLQAV	4	43.61	772.430	13.571	8.650	-7.6	-6.4
118	REGLQAV	4	43.74	772.430	-0.968	8.650	-7.5	-6.4
119	TVLTNEQLIEFA	4	43.88	1377.739	9.548	32.533	-8.4	-6.4
120	VNNDDRDSYR LQ	4	44.12	1494.692	-0.713	7.467	-7.8	-6.1
121	RQLMEAGLILA Y	4	44.22	1377.739	-12.132	32.533	-7.8	-6.7
122	AKQQQAV	4	44.3	772.430	-1.419	8.667	-7.8	-6.9
123	AQQKQAV	4	44.3	772.430	-1.419	8.667	-7.9	-6.8
124	FSVLHAPAF	4	45.08	988.527	2.085	10.800	-8.7	-8.2
125	IQAAQEL	4	45.1	772.431	14.282	6.650	-7.9	-6.7
126	ALPEEVIQH	4	45.32	1035.547	-0.031	16.233	-8.1	-6
127	ARDLQAV	4	45.4	772.432	0.716	8.683	-8.5	-5.8
128	ARDIGAAV	4	45.64	772.430	-1.445	8.667	-8.3	-6.7
129	AEVTI	4	45.99	532.298	0.930	15.117	-7.5	-6.4
130	LDKGIGTIISSPY	4	46.05	1363.743	-2.513	28.633	-7.8	-6.5
131	LNALPEEVIQH	4	47.51	1262.674	-0.101	23.117	-8.2	-6.4
132	LAGAAAGEL	4	47.75	772.431	14.253	6.600	-8.4	-6
133	VDLNGNHL	4	48.1	881.447	-1.072	13.600	-9	-6.1
134	RVFDGELQE	4	48.7	1092.532	-0.037	15.383	-7.6	-6.6
135	QSGNLL	4	49.4	631.344	4.762	15.950	-7.2	-6.2
136	NNEDTPVVAV	4	51.63	1057.516	0.069	19.050	-8.3	-6.6
137	DLNFTPR	4	51.76	862.440	-2.631	16.167	-9.3	-7.1
138	TFEEPQEQ	4	55.18	1007.432	0.169	9.400	-8.8	-7
139	TNDRPSIGNL	4	59.03	1086.554	-0.240	16.883	-7.8	-6.4
140	ITPEKNPQ	4	61.81	926.495	0.429	3.900	-8.2	-6.5
141	SVDINEGALLLP H	4	66.14	1377.734	-2.446	32.400	-8.6	-7
142	DFVLDNEGNPL	4	72.28	1232.580	0.816	33.650	-8	-6.7
143	FDNIVPm	3	39.61	851.380	-19.263	22.183	-8.5	-6.5
144	DVFRAIPSE	3	39.65	1033.532	1.049	21.417	-9.3	-6.8
145	LGVLTL	3	39.87	615.418	16.275	20.233	-7.4	-6.3
146	NRLLLTL	3	39.95	842.546	-0.399	31.567	-7.8	-6.9
147	QRVLLTL	3	39.95	842.546	-0.399	31.567	-8.3	-6.6
148	VINEGDANIE	3	40.17	1073.509	-1.429	12.433	-8	-6.3
149	QVARPS	3	40.26	657.368	0.310	4.083	-7.7	-6.3
150	VQARPS	3	40.26	657.368	0.310	4.083	-8.4	-7.1
151	WVGLGAAV	3	40.27	772.430	-6.821	8.667	-8.7	-6
152	LSIVDm	3	40.89	693.351	3.281	16.267	-7.4	-5.4
153	GLDLVSG	3	41.06	660.356	-0.391	20.483	-7.5	-6.1

154	FmHNGTLKE	3	41.08	1092.532	16.224	15.383	-8.3	-6.2
155	NTGDEPVVA	3	41.17	901.427	1.190	9.800	-8	-6.9
156	IQDKVTID	3	41.27	931.508	-1.728	11.083	-7.4	-5.8
157	IGDVLGS	3	41.77	660.356	0.055	20.517	-8	-7.3
158	EDAAILTQIPF	3	41.84	1217.652	8.698	27.550	-8.3	-6.7
159	ALVLPS	3	42.31	599.377	1.752	18.283	-8.1	-6.4
160	KGKIGm	3	42.37	649.367	-4.787	12.833	-6.5	-5.4
161	ESPGLKAA	3	42.9	772.430	12.935	8.667	-7.3	-6.7
162	KTNDTPmIGTL	3	43.48	1206.602	-1.562	18.500	-7.9	-7
163	PMVYPR	3	45.97	762.403	8.568	37.467	-9.2	-7.2
164	IIPLPVIKE	3	46.18	1021.665	-0.754	30.917	-7.8	-5.2
165	GHAPISLPNQ	3	46.69	1033.543	0.383	14.917	-8.4	-7.4
166	LPEGPAVKIG	3	47.15	980.576	-1.228	18.833	-8.1	-6.3
167	EMKEWLKE	3	48.7	1092.532	-6.810	15.383	-7.8	-5.7
168	KLGAGGF	2	39.6	649.367	0.295	12.900	-8.2	-7.2
169	SVNIIGQ	2	39.79	730.412	4.123	15.967	-7.9	-6
170	EGNLIGK	2	39.79	730.412	4.109	15.967	-7.3	-6.2
171	GENLIGK	2	39.79	730.412	4.109	15.967	-7.5	-6.7
172	LSKLKDLAE	2	39.96	1016.596	-2.933	21.133	-7.4	-5.3
173	AENNQRNFLA	2	39.97	1176.575	-0.814	12.400	-8.5	-7.4
174	VGQNLEL	2	39.99	772.431	13.773	6.617	-8.1	-5.7
175	FKGmSHLQE	2	40.33	1092.533	17.359	15.267	-8.1	-6.5
176	MLPSNIL	2	40.85	787.442	5.347	10.633	-7.5	-5.6
177	MLSPNIL	2	40.85	787.442	5.347	10.633	-7.5	-7.2
178	KAGLV	2	40.94	487.324	-0.320	5.350	-7.3	-6.2
179	GGESKPQFN	2	41.53	963.451	-2.130	5.267	-7.9	-6.9
180	VGSSKPQmN	2	41.53	963.451	-5.633	5.267	-7.8	-6.2
181	VEKEEGEY	2	41.56	982.445	8.781	13.933	-8.6	-7.2
182	KSLGGLE	2	41.61	703.398	-1.258	7.883	-7.7	-5.9
183	KSLNLE	2	41.61	703.398	-1.244	7.883	-7.8	-6
184	SMLAAVVVE	2	43.35	918.502	6.101	14.483	-7.3	-6.2
185	AImNILVE	2	43.62	918.502	6.106	14.483	-7.9	-6.6
186	SGFAPE	2	43.7	607.273	0.751	10.133	-9	-7.2
187	KISAGA	2	44.32	546.325	1.162	2.850	-7.9	-5.9
188	mALRVIAE	2	44.51	918.502	-6.131	14.483	-7.9	-5.8
189	KEQDQAAVESHRRAAAATASG	2	44.81	2154.045	-9.368	9.317	-6.7	-6.3
190	ASNLPHAS	2	46.2	796.394	-1.051	4.717	-8	-7.5
191	VNPESQQGSPR	2	46.31	1198.581	0.089	4.000	-7.5	-6.8
192	QSGGGII	2	46.82	631.344	4.746	15.950	-7.1	-5.8
193	VAEANVAV	2	47.22	772.432	15.254	8.683	-8.6	-6.7
194	YVVNPDNDE	2	47.64	1064.454	0.387	10.967	-8.6	-7.7

195	VKNNNPFSF	2	48.68	1066.530	-1.505	24.667	-8.8	-6.9
196	mKLEEARAE	2	48.7	1092.532	-3.135	15.383	-7.2	-6.4
197	GALFLYA	2	48.88	754.426	16.545	23.217	-9.5	-8
198	AGVQALA	2	49.54	629.361	-1.118	3.950	-8.4	-7.1
199	KGLAGGV	2	50.11	601.366	-1.334	7.833	-7.6	-6.1
200	RNTGNLL	2	51.95	787.444	2.142	10.600	-7.7	-6.5
201	NEGALLLPH	2	57.78	963.528	1.883	21.683	-8.2	-6.6
202	AILEQPISVS	2	74.04	1056.593	-0.259	25.800	-8.1	-6.6
